# A Transit Time-Resolved Microflow Cytometry-Based Agglutination Immunoassay for On-Site C-Reactive Protein Detection

**DOI:** 10.3390/mi12020109

**Published:** 2021-01-22

**Authors:** Jianxi Qu, Yushan Zhang, Mathieu Chenier, Chang-qing Xu, Lan Chen, Yonghong Wan

**Affiliations:** 1 School of Biomedical Engineering, McMaster University, 1280 Main Street West, Hamilton, ON L8S 4L8, Canada; quj@mcmaster.ca (J.Q.); zhang749@mcmaster.ca (Y.Z.); 2 Department of Engineering Physics, McMaster University, 1280 Main Street West, Hamilton, ON L8S 4L8, Canada; chenim1@mcmaster.ca; 3 Department of Pathology and Molecular Medicine, McMaster University, 1280 Main Street West, Hamilton, ON L8S 4L8, Canada; chenlan@mcmaster.ca (L.C.); wanyong@mcmaster.ca (Y.W.)

**Keywords:** C-reactive protein, microflow cytometry, agglutination immunoassay, on-site detection

## Abstract

An accurate and rapid microflow cytometry-based agglutination immunoassay (MCIA) suitable for on-site antibody or antigen detection was proposed. In this study, quantitative C-reactive protein (CRP) detection was chosen as a model assay in order to demonstrate the detection principle. The average transit time was employed to estimate the extent of the agglutination reaction and improve the detection accuracy as compared to the intensity-dependent methods. The detection time was less than 8 min. and only a 20 µL serum sample was needed for each test. The results showed a linear relationship between the average transit time of aggregates and CRP concentrations ranging from 0 to 1 µg/mL. The R^2^ of this relationship was 0.99. The detection limit of this technology was 0.12 µg/mL CRP. The system used for CRP detection can be extended to also monitor other clinically relevant molecules.

## 1. Introduction

Particle counting immunoassay is a sensitive and practical tool for the detection of antibodies and antigens. It is based on the principle that the agglutination reaction reduces the number of free particles in a solution. Unlike the latex fixation test from which it is derived, the particle counting immunoassay avoids the difficulty of determining the end point by the naked eye, avoids the risk of non-specific agglutination that is caused by other factors, has higher sensitivity, and it can be fully automated when used with a particle counter [1].

Flow cytometry is a top choice for particle counting and sizing in clinical diagnostics. Microflow cytometry is more competitive than the conventional flow cytometry, due to its low consumption of reagents, simple operation procedures, short assay time, and cost-efficiency [2]. The combination of particle counting assays and microflow cytometry makes on-site immunoassay detection possible. Microflow cytometry was first used for the measurement of aggregates that formed in a particle-based immunoassay in 2003. The intensity of scattering light was employed in order to measure the aggregates. However, deviations in the scattering light intensity were high, leading to a reduced accuracy [3]. The forward scattering light in microflow cytometry can also be applied to distinguish monomers from aggregates in a particle-enhanced immunoassay. The proportion of monomers is associated with the target analyte concentrations. Biotinylated bovine serum was detected in order to verify this method with a detection limit of 1.5 pM [4].

Enzyme-linked immunosorbent assay (ELISA) and turbidimetry are two common assay formats that quantify antibodies or antigens in practical applications. Although the ELISA is renowned for being highly sensitive, it requires well trained technicians, long incubation times, and elaborated procedures to perform, which complicates the development of its on-site applications. The assay time of antibody detection by ELISA must be shortened from a few hours to less than 30 min. to meet the requirement for on-site detection. Commercial analyzers are usually designed according to the particle agglutination turbidimetric immunoassay. In this method, functionalized particles are conjugated with specific antibodies, and the agglomerates form in the presence of analyte [5]. The sensitivity of this method is reduced by noise factors, such as dust or large agglomerates. Typically, analyzers that are based on turbidimetry are applied for the detection of high levels of analytes, especially when the requirement for the detection accuracy is not critical, such as differentiating bacterial and viral infections in clinical settings [6]. It is important to develop a technology that can realize quantitative antibody or antigen detection with a short assay time and high sensitivity for on-site diagnosis.

In this work, we report a transit microflow cytometry-based agglutination immunoassay (MCIA) for on-site and quantitative detection of antibodies or antigens in serum samples. C-reactive protein (CRP) detection is chosen as a model assay for verifying this method. CRP is a primitive acute phase protein that is an indicator of tissue damage and inflammation. The CRP levels can increase to more than 500 µg/mL after the onset of an acute-phase stimulus. The serous CRP level in healthy individuals is normally less than 10 µg/mL [7]. The median value of CRP in healthy young adults is 0.8 µg/mL. The 90^th^ and 99^th^ centile are 3.0 µg/mL and 10 µg/mL, respectively [8]. Elevated CRP levels are associated with many diseases, including angina pectoris [9], coronary heart disease [10], inflammatory bowel disease [11], H1N1 influenza [12], Parkinson’s disease [13], and metabolic syndrome [14]. The risk of occlusive arterial disease increases when the CRP levels rise above 3 µg/mL [15]. Unlike other biomarkers, the CRP levels are stable over a long period of time without obvious daily or seasonal changes. Accurate and rapid CRP detection can play an important role when evaluating the progression of disease; the CRP levels can be used to monitor the evolving health status of an individual and allow for informed treatment plans. The MCIA is suitable for CRP detection and it can be a complementary tool during diagnosis. The assay is based on the turbidimetric format. The aggregates, which are produced by the interaction of CRP and anti-CRP antibodies, are characterized by a microflow cytometry while using their transit time through an interrogation region. A linear correlation between CRP levels and the average transit time of reaction mixtures was established.

## 2. Materials and Methods

**Figure 1:**
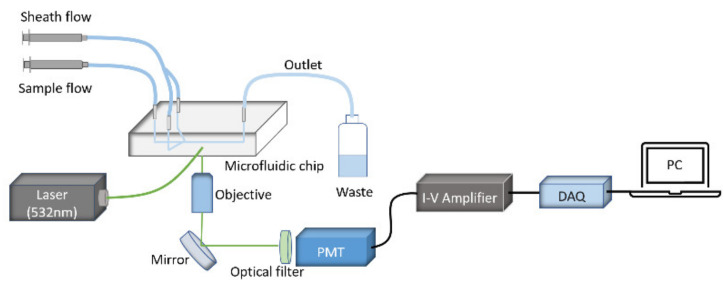
A schematic diagram of the microflow cytometry-based agglutination immunoassay (MCIA) setup.

The experimental setup of the microflow cytometry for CRP detection is shown in Figure 1, and more details of the fabrication process can be found in a previous publication by Benjamin et al. [16]. A green laser of 532 nm was coupled into a waveguide by an optical fiber. Subsequently, the coupled light was focused by an on-chip lens system to an interrogation region in a microfluidic channel. The reaction mixtures were injected by a syringe pump and then hydrodynamically focused in two dimensions by sheath flow into the center of the microchannel. Side scattering light was produced when the aggregates passed through the interrogation region and were detected by a photomultiplier tube (PMT). The converted electric current was amplified further to voltage signals by an I-V amplifier. A data acquisition (DAQ) device was used in order to digitalize the signals, which were recorded and analyzed by a custom program [17].

## 3. Procedure for Reaction

**Figure 2:**
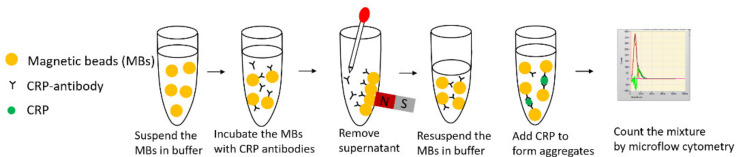
The principle of the MCIA.

Figure 2 displays a schematic illustration of the MCIA for CRP detection. The whole immunoassay was divided into three steps. Antibody coupling was the first step. Dynabeads M-270 Epoxy (Thermo Fisher Scientific,Waltham, MA, USA) magnetic beads (MBs) were coated with anti-CRP antibodies (PA1-29087, Thermo Fisher Scientific, USA) according to the user manual. The coated MBs were kept at 4–8 ℃ over a duration of one month while being preserved in sodium azide. The second step was to form aggregates. The coated MBs were diluted by phosphate buffered saline (PBS) to 20 µL with a final bead concentration of 1.0 × 10^7^ beads/mL. The diluted 20 µL MBs solution was mixed with 20 µL of CRP (Sekisui Diagnostics, Burlington, MA, USA) with gentle shaking or mixing with a pipette for 10 s. The mixture was incubated for 4 min., and aggregates formed during this process. This step can be manually performed in a centrifuge tube. PBS was used as a negative control. The final step was the detection of aggregates by the microflow cytometry. The resulting mixture was transferred from the microcentrifuge tube into the sample syringe pump for detection by the microflow cytometry. The sample flow rate and the sheath flow rate were set at 200 µL/hour and 800 µL/hour, respectively. Four min of raw data from side scattering (SSC) light signals were collected for analysis. 

## 4. Results and Discussion

**Figure 3:**
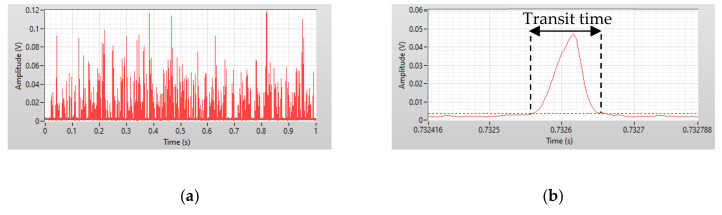
(**a**) One second of data recorded by the LabView program. (**b**) A detailed view of the pulse produced when particles passed through the interrogation region.

Figure 3a shows one second of data from a 0.50 µg/mL CRP test, which were recorded by a custom LabView program. The x-axis is the time in milliseconds and the y-axis is the amplitude of the pulses produced when the reaction mixtures pass through the interrogation region. Distinguishing between different sizes of aggregates using scattering light intensity is impossible, because the non-spherical nature of the aggregates can cause a wide scattering light intensity distribution and an irregular increase of intensity. Transit time was employed to solve this problem. In Figure 3b, the red solid line is the side scattering signal when one particle passes through the interrogation region, which is referred to as a count. The green dashed line is the threshold value that is the standard deviation of the background noise. The threshold was used to distinguish effective counts from noise. The interval between the intercepts of the threshold and the pulse is defined as the transit time.

In order to calculate the transit time of a particle passing through an interrogation region, the cross sectional area of the microchannel is defined as *S*, *L* is defined as the length of the interrogation region, *d* is the diameter of the MB, *n* is the number of MBs that form a signal aggregate, *Q* is the total flow rate, and *v* is the velocity of particles in the channel. The transit time, *τ*, is calculated by the following equation

(1) $$\tau =\frac{L+nd}{v}=\frac{L+nd}{Q/S}$$

*L* was 10 µm and *S* was 50 µm × 100 µm, according to the design of the microflow cytometry. Particles in the center of the channel have the maximum velocity, close to twice the average flow velocity due to laminar flow of the sample in the channel. For a single magnetic bead, the *τ*_min_ was calculated to be 115.2 µs. For dimers, the τ_min_ was 140.4 µs. When compared to a single bead, the difference in transit time that was caused by the agglutination of two beads was 25.2 µs.

**Figure 4:**
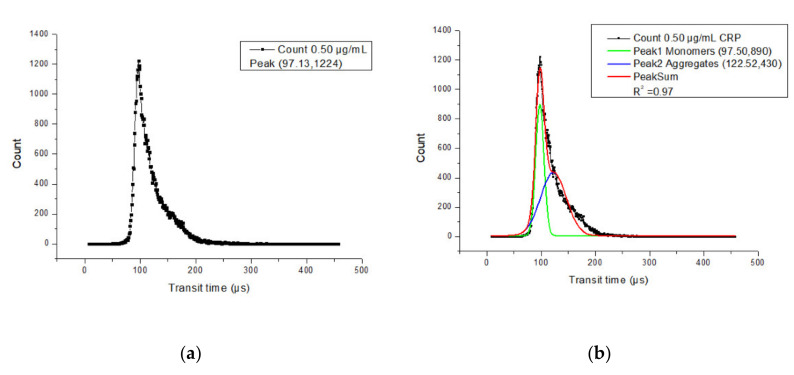
(**a**) Histogram of the transit time of all counts using a 0.50 µg/mL C-reactive protein (CRP) measurement. Each square was the count number of the corresponding transit time. (**b**) Histogram of the transit time of all counts using 0.5 µg/mL CRP with 2-order Gaussian fitting.

Most of the single beads have a transit time around 97 µs, as shown in Figure 4a. The distribution of the transit time between monomers and aggregates can be separated by Gaussian fitting, which is shown in Figure 4b. By the observation of aggregates through a microscope (Eclipse E200, Nikon, Mississauga, ON, Canada), most of the aggregates were dimers, so the number of peaks for Gaussian fitting was set as 2. The green solid line is the monomer distribution, which has a peak transit time of 97.5 µs, while the red solid line is the aggregate distribution, which has a peak transit time of 122.52 µs. The difference between these two peaks is 25.02 µs. Three 0.5 µg/mL CRP samples were tested and the difference in transit time was 25.00 ± 0.11 µs. The mean value of the difference is close to the theoretical calculation value of 25.20 µs. Although the transit times of both monomers and dimers during the experiment were smaller than the estimated theoretical calculations, the difference between experimental results and theoretical calculations is small. This means that the microflow cytometry is sensitive enough to discriminate between monomers and dimers by the transit time. The shorter transit time during the experiment may have been caused by a small cross-sectional area. When the cross-sectional area of the microchannel is smaller than 50 µm × 100 µm, the velocity of the particles in the channel will be larger, which causes the transit time to be less than the theoretical calculation. If the beam waist in the microchannel is smaller than 10 µm, the detected transit time will be less than calculated.

**Figure 5:**
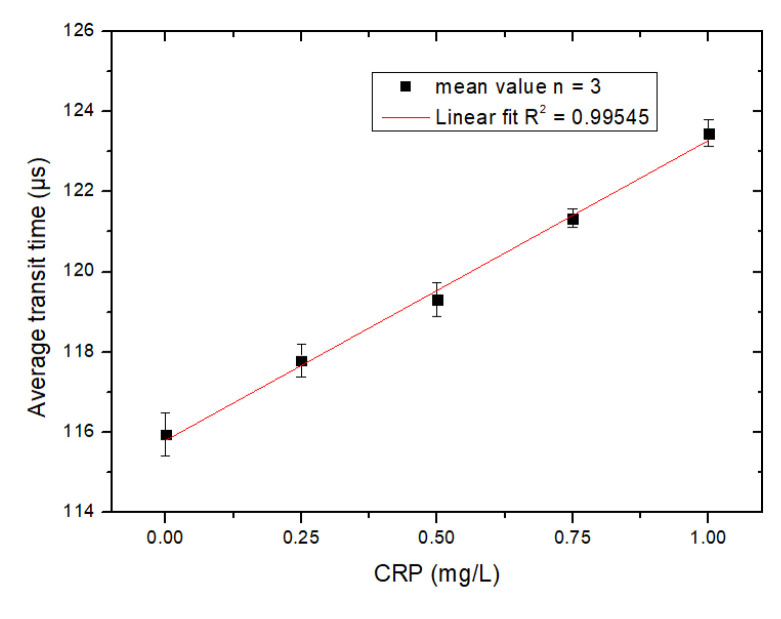
The relationship between the average transit time and CRP detected by the MCIA. Phosphate buffered saline (PBS) was used as the negative control. Each square and error bar were the mean value and standard deviation, respectively, of three parallel measurements.

The microflow cytometry that was used here was two-dimensional hydrodynamic focusing, so a small portion of particles were travelling at a lower velocity and had a longer transit time. In order to simplify data analysis, the average transit time was used to characterize the transit time distribution of all events. With higher concentrations of CRP in the sample, more aggregates formed, so the average transit time was higher than samples that contained less CRP and fewer aggregates. Figure 5 shows the relationship between the average transit time and CRP concentration measured by the microflow cytometry. The x-axis is the level of CRP and the y-axis is the average transit time. The average transit time increases as the CRP level increases. However, when the level of detected CRP is larger than 1.0 µg/mL, the proportion of aggregates in the sample decreases due to the Hook effect, which is an immunologic phenomenon: when the concentration of CRP is much higher than the antibody’s, the aggregate formation is impeded, due to the lack of binding sites for adjacent MBs, which would be occupied by excess CRP [18]. A ten-fold dilution of the serum sample is needed before detection in view of the fact that the healthy individuals have serous CRP level normally less than 10 µg/mL. The concentration of conjugated beads detected by the MCIA should be no less than 5.0 × 10^6^ beads/mL in order to ensure sufficient agglutination occurs during the antibody-antigen interaction. 

**Figure 6:**
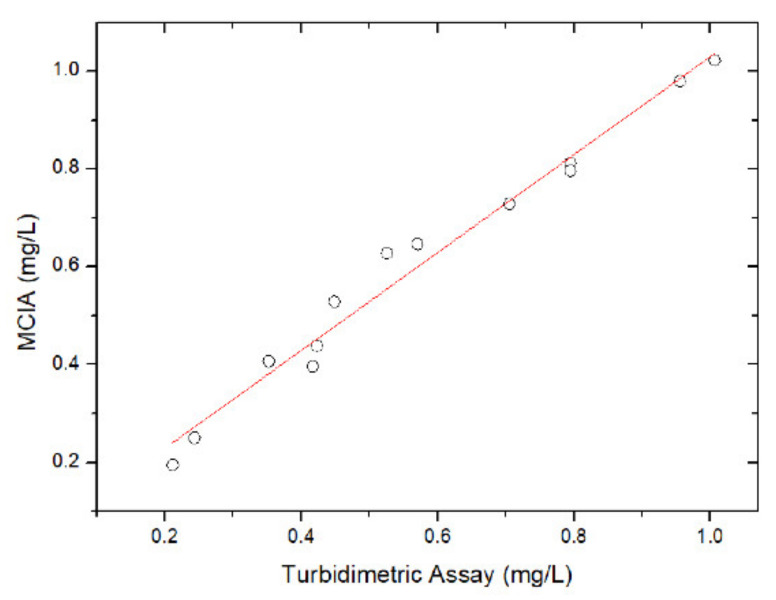
The correlation between the turbidimetric assay and the MCIA (n = 13).

The accuracy of MCIA was validated by assaying the same CRP samples by the MCIA and a turbidimetric immunoassay (Beckman DU 800 UV/Visible Spectrophotometer, Beckman Coulter Inc., Fullerton, California, USA). Figure 6 shows the correlation between the CRP concentration that was measured by the MCIA method and the conventional turbidimetric assay. A correlation coefficient r as high as 0.98 is achieved, which implies that accurate CRP concentration measurement can be achieved by the MCIA, as shown in Figure 6.

**Figure 7:**
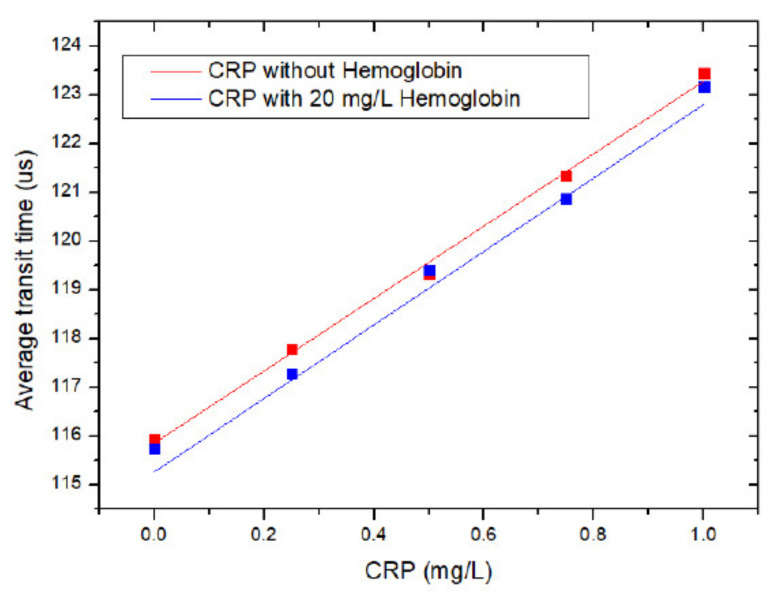
The interference of hemoglobin to CRP measurement.

Hemolysis is one of the most prevalent pre-analytical interferences and a main source of error during clinical tests [19]. The release of hemoglobin caused by hemolysis can contaminate serum samples. Hemoglobin (H7379, Millipore-Sigma, Oakville, ON, Canada) was added to CRP samples with a final concentration of 20 mg/L to test the interference of hemoglobin to CRP tests. Each sample was tested by the MCIA three times according to the procedure shown in Figure 5. The x-axis indicates the concentration of CRP, and the y-axis is the average transit time, as shown in Figure 7. The square block is the mean value of three parallel samples for each group. The red curve presents the transit times for CRP samples without hemoglobin and the blue curve shows the transit times for CRP tests with hemoglobin added. SPSS software (IBM) was used in order to compare slopes and intercepts of these two regression curves. The critical value was set as 0.05. The calculated p-value for the slope and intercept are 0.908 and 0.07, respectively. Both p-values are larger than 0.05, which suggests that there is no significant difference between these two curves and the interference of hemoglobin to CRP tests is acceptable.

## 5. Conclusions

A transit time resolved MCIA has been proposed and demonstrated while using CRP detection, with a detection limit of 0.12 µg/mL CRP. A linear correlation with a R^2^ of 0.99 has been achieved between CRP concentrations ranging from 0 to 1 µg/mL and the average transit time of the aggregates in the tested samples. The MCIA correlated well with the turbidimetric assay. It has been shown that the effect of hemolysis is negligible in the proposed method for CRP detection. The detection range of this method is, at most, 1 µg/mL CRP. The normal serous CRP level in healthy individuals is less than 10 ug/mL, so only a 10-fold dilution is needed for detection. The whole test can be broadly applied to other antigens by only replacing the antibody that is coated on the magnetic beads, which makes this testing method widely applicable for the quantitative measurement of other antigens or antibodies. Further investigation of this testing method should be done within an on-site clinical setting in order to investigate its efficiency.
